# Supplementary figures and images for: Adherence to the Mediterranean Diet in Spanish Population and Its Relationship with Early Vascular Aging according to Sex and Age: EVA Study
Source: Nutrients. 2020 Apr 8;12(4):1025. doi: 10.3390/nu12041025 (PMC7231158; doi:10.3390/nu12041025)

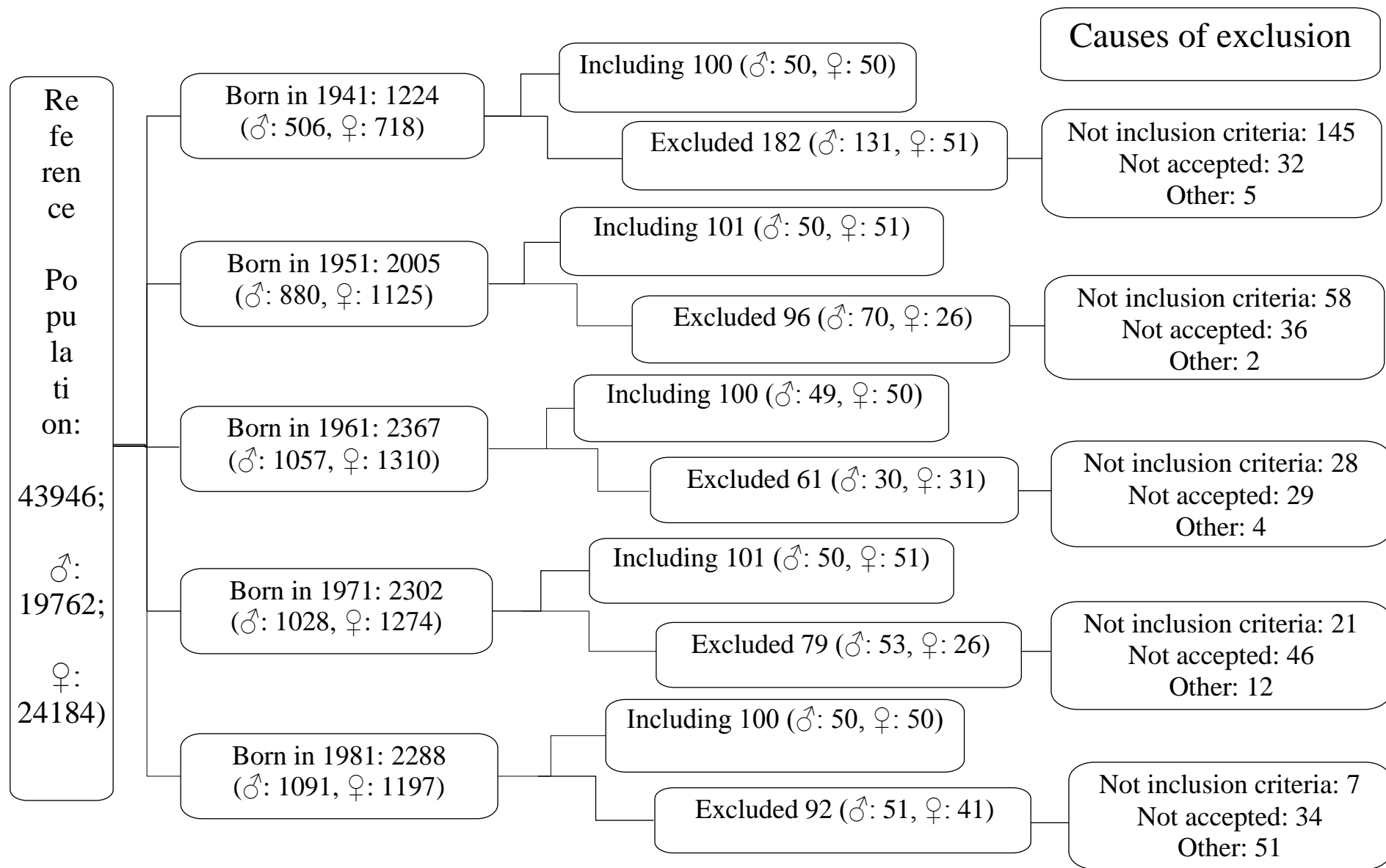

Figure S1: Flow diagram of early vascular aging study.

Supplement: Supplementary file 1 [file nutrients-12-01025-s001.pdf]
